# Model Predictive Temperature Control for Retinal Laser Treatments

**DOI:** 10.1167/tvst.13.9.28

**Published:** 2024-09-27

**Authors:** Viktoria Kleyman, Sophie Eggert, Christian Schmidt, Manuel Schaller, Karl Worthmann, Ralf Brinkmann, Matthias A. Müller

**Affiliations:** 1 Leibniz University Hannover, Institute of Automatic Control, Hannover, Germany; 2 University of Lübeck, Institute of Biomedical Optics, Lübeck, Germany; 3 Medical Laser Center Lübeck, Lübeck, Germany; 4 Technische Universität Ilmenau, Institute of Mathematics, Ilmenau, Germany

**Keywords:** retinal photocoagulation, hyperthermia, fluorescence measurements, model predictive control, extended Kalman filtering

## Abstract

**Purpose:**

Manual, individual adjustment of the laser power in retinal laser therapies is time-consuming, is inaccurate with respect to uniform effects, and can only prevent over- or undertreatment to a limited extent. Automatic closed-loop temperature control allows for similar temperatures at each irradiated spot despite varying absorption. This is of crucial importance for subdamaging hyperthermal treatments with no visible effects and the safety of photocoagulation with short irradiation times. The aim of this work is to perform extensive experiments on porcine eye explants to demonstrate the benefits of automatic control in retinal laser treatments.

**Methods:**

To ensure a safe and reliable temperature rise, we utilize a model predictive controller. For model predictive control, the current state and the spot-dependent absorption coefficients are estimated by an extended Kalman filter (EKF). Therein, optoacoustic measurements are used to determine the temperature rise at the irradiated areas in real time. We use fluorescence vitality stains to measure the lesion size and validate the proposed control strategy.

**Results:**

By comparing the lesion size with temperature values for cell death, we found that the EKF accurately estimates the peak temperature. Furthermore, the proposed closed-loop control scheme works reliably with regard to similar lesion sizes despite varying absorption with a smaller spread in lesion size compared to open-loop control.

**Conclusions:**

Our closed-loop control approach enables a safe subdamaging treatment and lowers the risk for over- and undertreatment for mild coagulations in retinal laser therapies.

**Translational Relevance:**

We demonstrate that modern control strategies have the potential to improve retinal laser treatments for several diseases.

## Introduction

Photocoagulation serves as an effective treatment for a variety of retinal diseases. Green-light laser pulses are (predominantly) absorbed in the retinal pigment epithelium (RPE), leading to denaturation of the tissue if the temperature is above a certain threshold.^1^ Depending on the disease, a different intensity of the coagulation is desired: strong coagulations to induce scars in order to prevent retinal detachment^2^ or mild coagulations to treat diseases such as diabetic retinopathy^3^ or diabetic macular edema.^4^ Besides coagulation, subdamaging treatments are under investigation that stimulate retinal metabolism by hyperthemia.^5^^–^^8^ Irradiation times are usually 30 to 200 ms, which is below the physician’s reaction time. However, as the light-scattering and retinal absorption vary within the eye,^9^ manual adjustment of the laser power at each irradiation spot would be necessary for the same effect. This titration procedure is time-consuming, and prevention from overtreatment or undertreatment is only possible to a certain extent.^10^ For reliable subdamaging treatments, it is inevitable to have additional information (e.g., about the temperature at the irradiated spot). Otherwise, due to a lack of visibility, the ophthalmologist cannot determine whether the treatment was successful. The measurement of an average depth-weighted temperature at the irradiated spot^11^ allows for temperature feedback and, hence, control strategies toward safer and faster treatment for a variety of diseases.

In recent years, various control strategies for photocoagulation have been developed.^12^^–^^15^ In Baade et al.^14^ and Schlott et al.,^15^ different open-loop strategies are presented, whereas Abbas et al.^12^ and Herzog et al.^13^ proposed closed-loop control approaches. All methods are based on controlling the hottest temperature at the irradiated spot, that is, the peak temperature in the center of the RPE. To this end, the measured depth-weighted temperature is translated to the peak temperature by means of an offline identified conversion function.^16^ This function is only valid under some assumptions (e.g., constant laser power or constant relation of the absorption coefficients between different tissue layers), and might lead to errors in the approximation of the peak temperature (if the assumptions are not met). The proportional-integral-derivative (PID) controller design^12^^,^^13^ is based on a linear first-order model. The (unknown) parameters (i.e., the static gain and the time constant) were identified offline. Due to patient and spot-dependent tissue parameters, such as the absorption coefficients, a wide parameter range needs to be taken into account to design the controller. This may lead to a lack of performance because the controller gains need to be robust against the whole parameter range. Furthermore, bounds on the peak temperature, which are crucial for safety reasons, cannot be incorporated in the proposed schemes.^12^^,^^13^ Nevertheless, first preclinical experiments based on the PID controller with anesthetized rabbits show that closed-loop control can improve retinal laser treatments.^17^

To ensure a safe and efficient treatment, we developed a model predictive control (MPC) strategy for retinal laser treatment.^18^ This proposed method includes four major differences to the aforementioned approaches: (1) modeling of the heat diffusion during laser irradiation by a partial differential equation,^19^ (2) conducting a parametric model order reduction to obtain a (parameter-dependent) low-dimensional model,^19^^,^^20^ (3) designing a suitable observer for joint (online) state and parameter estimation,^21^^,^^22^ and, finally, (4) designing a model predictive controller that is suitable for retinal laser treatments.^18^ In doing so, we can overcome the drawbacks of previous controllers in the following way. First, by modeling the heat diffusion, we can consider the peak temperature as one output of the system and the volume temperature as another one without approximating any conversion function. Second, estimating the absorption coefficient allows for an adaptive (and hence in general better performing) controller. Third, limits on the laser power and peak temperature can be implemented in the optimal control problem, which improves the safety of closed-loop control. We found that an extended Kalman filter (EKF) is a suitable estimator for our purposes.^22^ Therefore, we proposed an EKF-MPC approach in Schaller et al.^18^ for temperature-controlled retinal laser treatments, where also some first proof-of-concept closed-loop experimental results were reported.

The contribution of this article is as follows. First, we conduct extensive experiments in open loop and closed loop to allow for statistical evaluation of the proposed EKF-MPC framework in terms of different measures such the duration of overshoot, the time to reach the desired temperature, and others. Second, as a main contribution of this work, we validate our control framework by means of fluorescence measurements. In particular, all experiments were combined with cell vitality stains (calcein-AM) to determine the cell damage. The resulting fluorescence images were then used to measure the lesion size caused by irradiation. To validate the accuracy of the (peak temperature) estimation via EKF, we compare the estimated peak temperatures and cell damage with the known temperature threshold of (RPE) cell death, that is, 53°C ±2°C.^1^ We show that the proposed technique is especially suited for nondamaging, invisible treatments as the peak temperature is estimated reliably, and consequently, undesired coagulation or insufficient heating can be avoided. Furthermore, a major objective of the proposed closed-loop control is to reduce the variation in lesion size for mild coagulation compared to the case where a constant open-loop laser power is applied. This is achieved by aiming for the same peak temperature independent of the absorption.

This article is organized as follows. In the second section, we present the experimental procedure and the underlying model for estimation and control. Further, we (briefly) explain the joint state and parameter estimation and the model predictive controller used in this work. In third section, we show experimental open-loop and closed-loop results with a detailed analysis. Last, we conclude with a summary and an outlook with respect to future work.

## Materials and Methods

In this section, we present the experimental setup and the measurement procedure for the conducted experiments. After that, we briefly describe the modeling, the employed EKF, and the model predictive peak temperature controller.

### Experimental Setup

**Figure 1.**
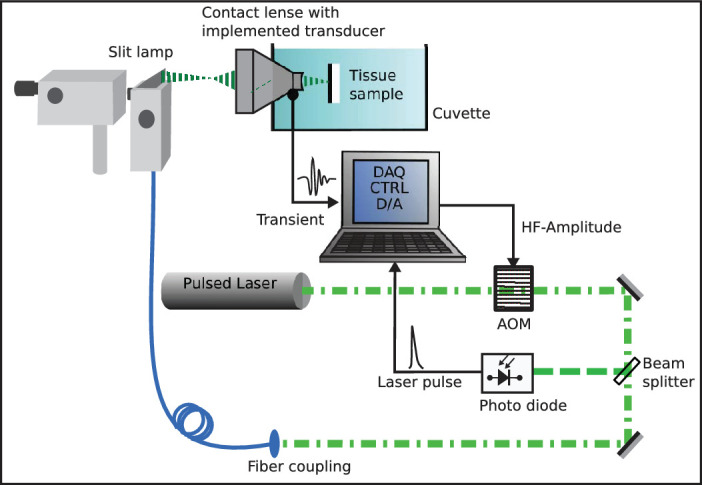
Experimental setup.

Figure 1 shows a sketch of the experimental setup. The beam of a frequency doubled diode pumped solid state Nd:YLF laser (2ENLPPLM90-005; Monocrom, Barcelona, Spain) with a wavelength of λ = 527 nm and a pulse duration of τ = 53 ns is aimed through an acousto-optic modulator (AOM) (R23080-3-LTD; Gooch & Housego, Ilminster, England, UK) to adjust and control the laser pulse energy. A small amount of the beam (≈0.5%) is coupled to a photo diode (PDA10A2; Thorlabs Inc., Newton, New Jersey, USA) to measure the laser pulse energy and compensate for energy fluctuations. The remaining beam is coupled to an optical fiber with a core diameter of *D* = 50 µm and numerical aperture of *NA* = 0.1. After leaving the slit lamp, the laser beam is imaged onto the tissue sample by means of an ophthalmic contact lens (Mainster Focal Grid; Ocular Instruments, Bellevue, Washington, USA) with a spot diameter *D* = 200 µm on the sample’s surface. The contact lens was customized with a ring-shaped piezo-ceramic transducer (Medical Laser Center Lübeck, Lübeck, Germany) as a pressure sensor and is attached to a sample cuvette filled with sodium chloride solution of 0.9%. The pressure transient depends on the laser pulse energy and the tissue’s temperature. A depth-weighted average temperature can be calculated as in [Ref. 11, Eq. 9] if the pressure transient and the pulse energy are known. A detailed description on the temperature determination can be found in Brinkmann et al.^11^ and Mordmüller et al.^23^ Both pressure transient and laser pulse signals are recorded by a fast data acquisition board (DAQ) (ADQ14; Teledyne SP Devices, Linköping, Sweden) and processed with C/C++ software.

The laser is operated with a pulse repetition rate of 10 kHz. Each millisecond the temperature is updated, which allows for estimation and control with a sampling rate of 1 kHz. All experiments are conducted on explants consisting of RPE, choroid, and sclera of enucleated porcine eyes with removed retina.

### Explant Preparation

The porcine eyes were collected from a local slaughterhouse, cooled until preparation, and used typically within few hours after enucleation. A scalpel was used to dissect the eyes and excess tissue was removed. A sample with diameter of 12 mm was punched out from the periphery of the fundus to avoid parts with the optical nerve. The retina was removed carefully with forceps. If necessary, the sample was rinsed with fresh sodium chloride solution to help the process. The RPE/choroid/sclera samples were stored in 0.9% sodium chloride solution in a fridge at 7°C until use. The sample was mounted in a cuvette filled with fresh sodium chloride solution at room temperature. Then, we waited 15 minutes in order for the samples to reach room temperature before we conducted the experiments. After the laser treatment, the samples were rinsed with fresh sodium chloride solution before three drops of the fluorescent dye calcein-acetoxymethylester were applied. The samples were left covered for 10 minutes before they were rinsed with sodium chloride solution again to remove the excessive dye. The fluorescence images were taken directly after, with a fluorescence microscope (Nikon eclipse T1, Tokyo, Japan). The eyes were used 4 to 6 hours postmortem.

### Measurement Routine

We perform two different experiments for a variety of eyes and spots. For the first experiment, an a priori chosen constant laser power is applied during the whole treatment phase (i.e., an open-loop control). For the second experiment, a (time-varying) laser power is set by the model predictive controller based on the current system’s state (i.e., a closed-loop control). We conducted 100 open-loop experiments from 5 different eyes and 356 closed-loop experiments from 12 different eyes. We evaluated 377 spots and excluded 79 spots due to corrupted data. We excluded data with outliers (i.e., a sudden, short-time temperature peak that is far higher than it can be explained by physics). Furthermore, we excluded data with a sudden drop in the measured temperature. As this occurred especially at high laser powers (in open loop) or high aim temperatures (in closed loop), we believe that the reason is strong coagulation inducing tissue shrinkage and thus spot translation. Our measurement approach is not suited for strong coagulation, which we will explain in more detail in “Closed-Loop Results.” At each spot, the (constant) open-loop input or the closed-loop input is applied to the system for 100 ms.

**Figure 2.**
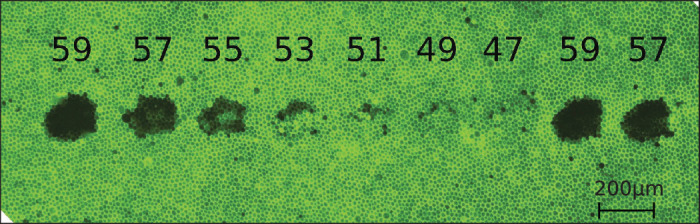
Microscope image of a fluorescent sample with dead cells (in black) and green-fluorescent living cells for different aim temperatures from 47°C to 59°C .

**Figure 3.**
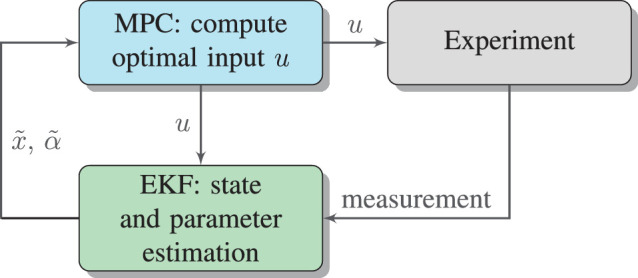
Estimation and closed-loop control scheme for retinal laser treatment.

The cell viability is visualized with calcein-acetoxymethylester (calcein-AM) (Thermo Fisher Scientific, Waltham, Massachusetts, USA). In living cells, the nonfluorescent dye calcein-AM is converted to green-fluorescent calcein. This allows distinguishing between living and dead cells after laser treatment. Using a fluorescence microscope, the cell damage caused by laser light can be measured by means of the lesion size, as shown in Figure 2. This allows for validation of the employed EKF-MPC approach. A scheme of the control loop is shown in Figure 3.

### Model

In this section, we briefly describe the mathematical model employed for estimation and control design. For further details, we refer to Kleyman et al.^19^^,^^22^

To describe the heat distribution inside the tissue during laser irradiation, we model five different layers of the eye fundus, namely, the retina, the RPE, the unpigmented part (Bruch’s membrane), the choroid, and the sclera with thicknesses according to [Ref. 22, Table 1]. The laser light initiates a temperature rise and thus serves as the heat source. The light–tissue interaction is modeled using Beer’s law^24^ and depends nonlinearly on the absorption at each spot. The absorption of laser light with a wavelength of 527 nm mainly takes place in the RPE and choroid^25^; hence, we consider absorption in these two layers with µ = [µ_rpe_, µ_ch_]^⊤^ for the RPE and the choroid. As the absorption is spot-dependent, we introduce an absorption parameter α = [α_rpe_, α_ch_]^⊤^ to scale the absorption, that is, µ = αµ_0_ with µ_0_ = [120,400, 27,000] m^−1^ from [Ref. 20, Table 1]. In this way, only the unitless absorption parameter needs to be estimated at each spot. To explain the fundamentals of the model used for control, we denote by *x*(*t*, ω) the temperature difference with respect to the ambient temperature at time *t* and spatial coordinate ω and the laser power at time *t* by *u*(*t*). Then, by means of the abovementioned Beer’s law, we get the heat equation

(1) $$\begin{array}{ccc} & & \rho C_{p}\frac{\partial x(t,\omega )}{\partial t}-k\Delta x\left(t,\omega \right)=\\ & & \;u\left(t\right)\frac{\chi_{I}\left(\omega \right)}{\pi R_{I}^{2}}\mu \left(\omega_{3}\right)e^{-\int_{0}^{\omega_{3}}\mu \left(\zeta \right)d\zeta }\; \end{array}$$

on a cylindrical domain with an inner cylinder modeling the irradiated region and an outer cylinder that represents the surrounding tissue. Here, χ_*I*_(ω) ∈ {0, 1} is the characteristic function of the domain that is heated by the laser, that is, χ_*I*_ = 1 inside the irradiated region. We choose the outer cylinder large enough such that the temperature increase at the boundary is negligible during typical treatment duration. Hence, we consider homogeneous Dirichlet boundary conditions and a homogeneous initial condition due to the fact that the eye explants are at room temperature. For the values of the positive parameters, *C*_p_, *k*, *R*_I_, we refer to [Ref. 20, Table 1]. Whereas the left-hand side of Equation (1) models the heat diffusion in the tissue, the right-hand side accounts for the amount of heat absorbed within the interval [0, ω_3_] by means of the exponential function. Leveraging rotational symmetry [Ref. 26, Section 2.2], the output corresponding to the volume temperature is defined by

$$\begin{array}{c} T_{\mathrm{vol}}\left(t\right)=\int_{z_{b}}^{z_{e}}x_{\mathrm{mean}}\left(t,\omega_{3}\right)\mu \left(\omega_{3}\right)e^{\int_{0}^{\omega_{3}}\mu \left(\zeta \right)d\zeta }\;d\omega_{3}, \end{array}$$

where *x*_mean_(ω_3_) is an average of the (two-dimensional) heat distribution at depth ω_3_ and *z*_b_, *z*_e_ denote the beginning and the end of the considered region. Correspondingly, the peak temperature is defined via

$$\begin{array}{c} T_{\mathrm{peak}}\left(t\right)=\max_{\omega }x\left(t,\omega \right). \end{array}$$

Numerical simulation showed that the hottest point, that is, the maximizer of the expression in the definition of the peak temperature *T*_peak_ lies in the center of the RPE layer, except for a very short initial phase. The coagulation and hence the visible damage (lesion size) is expected to start at the hottest point inside the tissue. Thus, the peak temperature can be considered as the threshold temperature for thermal denaturation. As mentioned before, the peak temperature *T*_peak_(*t*) cannot be measured. However, we consider *T*_peak_(*t*) as an additional output to allow for peak temperature control.

**Table 1.** Mean and Deviation of Lesion Size for Constant Laser Power in Open Loop.

*P*_*L*_	${\bar{d}}_{\mathrm{lesion}}$ [µm]	σ_d_ [µm]	Co. Var. *c*	No. Spots
25	99	48	0.48	20
30	137	52	0.38	16

After dimension reduction using rotational symmetry^26^ and spatial discretization of the heat diffusion Equation (1), a high-dimensional state-space model is obtained that is too large for real-time control in 1 kHz. Therefore, a low-dimensional surrogate model of order *n*_*x*_ = 6 is obtained by a parametric model order reduction (pMOR) that preserves the parameter-dependency as described in Schaller et al.^20^ Temporal discretization yields the discrete-time surrogate model in state-space

(2) $$\begin{array}{ccc} x(k+1) & = & Ax(k)+b(\alpha )u(k),\\ y(k) & = & C\left(\alpha \right)x\left(k\right)=\left(\begin{array}{c} c_{\mathrm{vol}}\left(\alpha \right)\\ c_{\mathrm{peak}} \end{array}\right)x\left(k\right),\; \end{array}$$

with $A\in R^{n_{x}\times n_{x}}$, $x\in R^{n_{x}}$, $b\left(\alpha \right)\in R^{n_{x}}$, $C\left(\alpha \right)\in R^{2\times n_{x}}$,  *y*(*k*) = (*T*_vol_(*k*), *T*_peak_(*k*))^⊤^, time instant *k*, and input *u*(*k*) that is the laser power.

Concluding this modeling part, we note that for linear heat equations with constant coefficients, analytical solution formulas are available using, for example, a Fourier ansatz^27^ or the Gaussian heat kernel.^28^ However, to evaluate these formulas, truncation of the Fourier series or an approximation of the convolution integrals is necessary. Thus, in this application, we utilize the sophisticated framework of finite differences methods combined with model order reduction to obtain a discrete-time model that can be efficiently evaluated and allows for optimal control in real time.^18^

### State and Parameter Estimation

To (online) estimate the states *x* and the absorption parameter α in a joint fashion, we extend the state-space model Equation (2) by adding α as an additional state with constant dynamics, that is, α(*k* + 1) = α(*k*) to the state-space model. As this yields a nonlinear model, we employ an EKF for estimation. The EKF is a well-known state estimator for nonlinear systems that is based on successive linearization at each time step (see, e.g., Chui and Chen^29^). A thorough evaluation of the EKF (in comparison with moving horizon estimation) in the context of retinal laser treatment can be found in Kleyman et al.^22^ The estimation of both absorption parameters α_rpe_ and α_ch_ is difficult for various reasons. First, the input needs to be exciting enough to allow for estimation of both parameters. This is especially difficult to achieve in closed-loop identification. Second, convergence can, in general, not be guaranteed in a joint state and parameter estimation resulting in biased estimates.^30^ However, for the estimation of only one absorption parameter, a constant input is exciting enough and convergence was reached in all cases in Kleyman et al.^22^ As the influence of the RPE absorption on the peak and volume temperature is higher than the influence of the choroidal absorption,^20^ only α_rpe_ is estimated. The absorption parameter in the choroid layer α_ch_ is chosen as the (constant) mean value that was found in [Ref. 20, Table 2].

### Peak Temperature Control

We aim for controlling the peak temperature as the coagulation is expected to start from there (as mentioned above). We assume that the lesion size can be scaled by controlling the peak temperature and that the same peak temperature results in a similar lesion size. To increase the safety of the treatment, a model predictive controller is employed that allows to incorporate (hard) input and output constraints by design.^31^^–^^33^ MPC predicts the future behavior of the system over a finite horizon N∈N to obtain the optimal input trajectory $u=\left(u_{k|k},...,u_{k+N-2|k}\right)\in R^{N-1}$. Here, *u*_*i*|*k*_ denotes the optimal input for time *i* (with *k* ⩽ *i* ⩽ *k* + *N* − 2) predicted at current time *k*. The optimal control problem (OCP)

(3) $$\begin{array}{c} \;\;\;\;\;\begin{array}{cc} \min_{u}\sum_{i=0}^{N-1}{\left|c_{\mathrm{peak}}x_{i|k}-T_{\mathrm{aim}}\right|}^{2} & \\ s.t.\;x_{i+1|k}=Ax_{i|k}+b\left(\tilde{\alpha }\right)u_{i|k} & i=k,...,k+N-2\\ x_{k|k}=\tilde{x} & \\ 0\leq u_{i|k}\leq P_{L,\max} & i=k,...,k+N-2\\ c_{\mathrm{peak}}x_{i|k}\leq T_{\max} & i=k+1,...,k+N-1. \end{array}\; \end{array}$$

is solved at each time instant *k* and the first (optimal) input *u*_*k*|*k*_ is applied to the system until the next time step. Then, the horizon is shifted and the OCP is solved again to obtain *u*_*k* + 1|*k* + 1_. This procedure is repeated until the end of the treatment. To avoid undesired side effects caused by (peak) temperature overshoots during control, the maximum allowed peak temperature *T*_max_ is constrained to 2°C above the aim temperature *T*_aim_, that is, *T*_max_ = *T*_aim_ + 2°C. As there are physical limits on the laser power, hard constraints on the maximum laser power *P*_L, max_ are included. At each time step, the initial condition for the state predictions, *x*_*k*|*k*_, and the parameter vector are set to the current EKF estimates $\tilde{x}$ and $\tilde{\alpha }$, respectively. A short horizon *N* = 5 is used so that the OCP can be solved in less then 1 ms. The optimization problem Equation (3) is solved by means of a C++-implementation with the solver OSQP.^34^ Further details and a in-depth comparison concerning different cost functions, horizon lengths, and sampling rates can be found in Schaller et al.^18^

## Results and Discussion

In this section, we show the experimental results for the conducted open-loop and closed-loop experiments. We evaluate the EKF by means of estimated peak temperatures, estimated absorption coefficients, and lesion sizes. The combined EKF-MPC approach is analyzed with regard to controller performance and lesion size. In addition, we carry out further simulations to estimate the error caused by constant assumed choroidal absorption.

### Open-Loop Results

The aim of the open-loop experiments is to verify if the EKF estimates reasonable states and absorption parameters that are used as initial values $\tilde{x}$ and $\tilde{\alpha }$ in the optimal control problem Equation (3). As the reduced states *x* in Equation (2) do not have a physical interpretation, we concentrate on the estimated peak temperature (i.e., the second output of Equation (2)) as a measure to assess the functionality of the observer. Moreover, the peak temperature has a major influence on the lesion size that is used to further verify the EKF.

**Figure 4.**
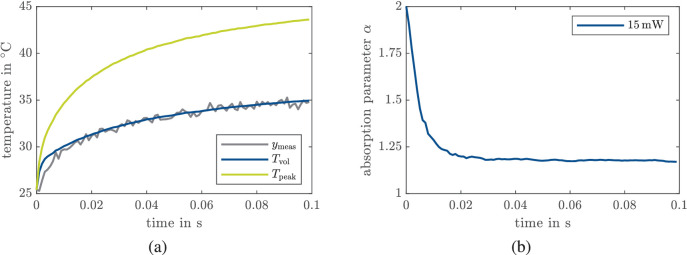
Measurement and estimation of a spot with a laser power of 15 mW and no visible damage. (a) Measured temperature *y*_meas_ (gray), estimated peak temperature *T*_peak_ (green), and estimated volume temperature *T*_vol_ (blue). (b) Absorption parameter estimation.

**Figure 5.**
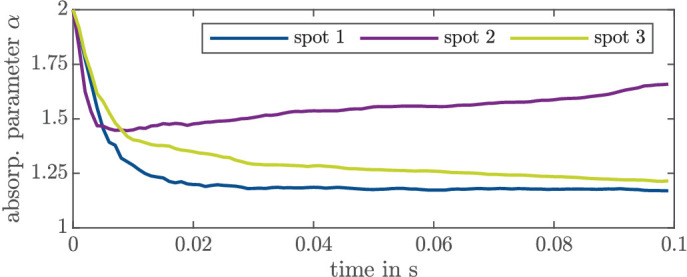
Estimated absorption parameter α for three different spots.

In Figure 4a, the measured volume temperature *y*_meas_ and estimated peak temperature *T*_peak_ are shown for one spot where a laser power of 15 mW for 100 ms was applied. We observe that α converges after approx. 20 ms to a constant value, as shown in blue in Figure 4b as a representative example of the convergence speed. However, for other spots, the parameter estimation shows a positive drift (after some settling time), as depicted in Figure 5 in purple or a negative drift as depicted in green. Note that these are different spots, and hence it is not expected that one obtains convergence to the same value.

**Figure 6.**
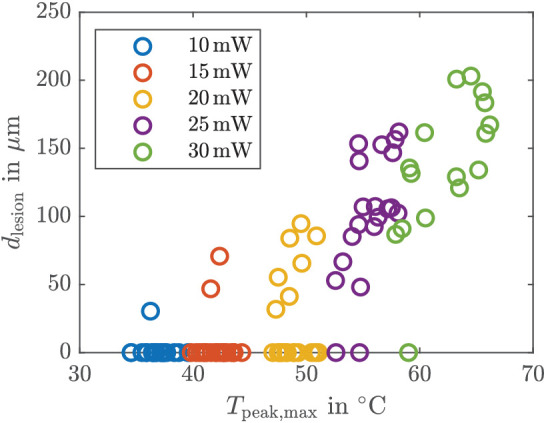
Estimated maximum peak temperature and diameter of lesion for constant laser powers.

In open-loop control with constant laser power, a desired temperature increase cannot be chosen a priori as in the closed-loop approach. Nevertheless, we can utilize the estimation of the peak temperature for validation of the EKF. To this end, we determine the maximum estimated peak temperature at each spot and compare it to the lesion size. Due to the open-loop approach, the temperature increases until the end of the treatment as visualized in Figure 4a. Figure 6 shows the maximum (estimated) peak temperature *T*_peak, max_ at the end of the treatment and the corresponding measured lesion diameter *d*_lesion_ caused by laser powers of 10 mW (blue), 15 mW (orange), 20 mW (yellow), 25 mW (purple), and 30 mW (green). For peak temperatures below 48°C, there is no visible lesion except for some outliers. For peak temperatures above 48°C, the lesion size increases with increasing peak temperature. For a constant laser power *P*_L_ = {25, 30} mW over 100 ms, the mean of the lesion diameter ${\bar{d}}_{\mathrm{lesion}}$, the standard deviation σ_d_, and the coefficients of variation $c=\frac{\sigma_{d}}{{\bar{d}}_{\mathrm{lesion}}}$ are shown in Table 1. The values for *P*_L_ ⩽ 20 mW are not presented as there was no visible lesion for most of the spots (cf. Fig. 6).

### Discussion of Open-Loop Experiments

In the following, we discuss the insights obtained from the open-loop experiments. In Figure 4b, the parameter converges to 1.2, which corresponds to an absorption coefficient in the RPE that is close to the value determined in Hammer et al.^35^ However, we consider a 10-times smaller value in the choroid layer compared to Hammer et al.,^35^ according to the value that we identified in Schaller et al.^20^ A recently published article^36^ that identified the absorption for the RPE and choroid together (as one part of the whole eye) suggests that the absorption is in the same order of magnitude as our (offline identified) values.^20^ The parameters that are estimated online via the EKF are in a similar range as in Regal et al.^36^ and Schaller et al.,^20^ which indicates that the online parameter identification works reliably. This was also shown in Kleyman et al.^22^ for a slightly different setup.

We now discuss the outcomes of the calcein measurements as reported in Figure 6 and Table 1. First, we notice that the estimated peak temperature in Figure 6, at which visible damage starts, closely matches the value reported in Denton et al.,^1^ where the threshold temperature in laser-induced damage was investigated. The authors found out that cell death starts at 53°C ±2°C for an irradiation duration between 100 ms and 1 s. These values are close to the estimated peak temperature at which visible damage starts in Figure 6, which leads to the conclusion that the employed EKF estimates reasonable peak temperatures and absorption parameters, which is of crucial importance for a reliable close-loop control. A possible reason why in Figure 6, in some (few) cases, lesions are already visible at temperatures below 50°C is the fact that the same (constant) choroidal absorption coefficient is used for all spots, which can lead to a small inaccuracy in the peak temperature estimation.

Furthermore, Figure 6 shows that the lesion size increases with increasing peak temperature, which supports our assumption that the lesion size is (mainly) influenced by the peak temperature. The strong deviation in the lesion size (in Fig. 6 and Table 1) for the same laser power is to be expected and (mainly) caused by varying tissue parameters, especially the absorption.^13^^,^^35^ In vivo experiments with chinchilla gray rabbits^37^ have shown a larger standard deviation for open-loop measurements as found here. This can be explained by additional influencing parameters like the light scattering within the eye, as in the case of explants consisting of RPE, choroid, and sclera only. An open-loop control cannot react to varying parameters as there is no feedback of the current system’s state by means of measurements, and hence, no knowledge about spot-dependent parameters can be incorporated. Spot-dependent tissue parameters lead to different peak temperatures and, thus, to different lesion sizes. Therefore, the lesion size can only be influenced very roughly by open-loop control. However, the lesion size also varies for similar peak temperatures. One reason could be that convergence is not always reached, as shown in Figure 5. As we would expect convergence much faster, we will discuss the influence of a drifting parameter in the following.

**Figure 7.**
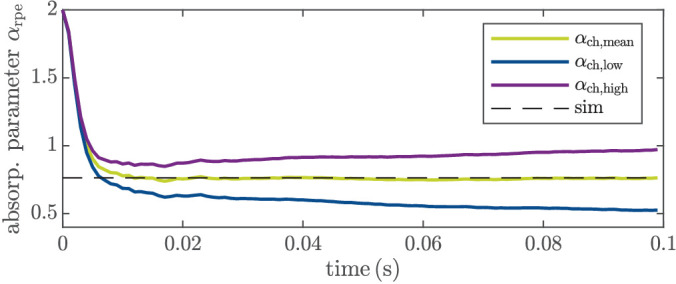
Estimated absorption parameter α_rpe_ for different α_CH_ in the plant model, mean value (green), lower bound (blue), upper bound (purple), and α_rpe_ of the plant as reference (black, dashed).

The slight drift could be caused by different reasons, such as different tissue parameters (than assumed in the model) or changes in the tissue during irradiation that influence the temperature measurement. To further examine this issue, we conducted additional simulations where we used the full-order model as plant model and where the reduced model Equation (2) used by the EKF has different tissue parameters (i.e., different thickness of the layers and diffusion properties *C*_p_, *k*, ρ) and/or different choroidal absorption parameters α_ch_. When using different tissue parameters, we observed some offsets for the identified parameters, but there was no drift (except for a very slight one in case of a different thermal conductivity *k*). On the other hand, a different choroidal absorption parameter α_ch_ in the reduced model (compared to the “real” one, i.e., the one used in the full-order model) indeed can result in a parameter drift and very slow convergence after several hundred milliseconds to a false parameter value. In particular, we considered in the full-order plant model a lower and a higher choroidal absorption parameter α_ch, low_ and α_ch, high_, respectively. We choose α_ch, low_ and α_ch, high_ such that 95% of the identified absorption parameters α_ch_ of the case study in Schaller et al.^20^ are within these values, that is, α_ch, low_ = 0.04 and α_ch, high_ = 0.15. If α_ch_ is higher, the tissue will heat up more intensely, which has also an influence on the volume temperature. If the volume temperature is higher than expected (by the EKF model), the estimated absorption parameter α_rpe_ will compensate for this. Figure 7 shows the estimated absorption parameters α_rpe_ for different choices of α_ch_ in the full-order plant model. In the EKF, α_ch_ is set constant to the mean value α_ch, mean_ from Schaller et al.,^20^ which means that there is a mismatch between the plant and the EKF model. If α_ch_ is higher than assumed by the EKF, there is a negative drift of α_rpe_ as depicted in blue and for the opposite case (α_ch_ is smaller than assumed) in purple. Based on these simulations, we conclude that the drift is mainly caused by a different absorption in the choroid. This conclusion is strengthened by the fact that the drift is spot-dependent as well as the absorption in the choroid. Independent of the cause, the drift has an influence on the estimated peak temperature and therefore on the closed-loop performance, as we will discuss in “Discussion of Closed-Loop Experiments.”

A major objective of our closed-loop control is to reduce the variation in lesion sizes compared to the case where a constant (open-loop) laser power is applied. To this end, we aim for the same peak temperatures independent of the absorption yielding a precise treatment. This is only possible in closed-loop control where the aim temperature can be set to a certain value. Then, the input signal (laser power) depends on the individual spot properties, which are estimated by the EKF. Furthermore, a certain peak temperature can be reached faster in closed loop since it can react to different absorption coefficients (by different laser powers) and therefore different temperature increases. This can reduce the duration of treatment. In open loop, the laser power can only be set before treatment, without knowing whether and when a desired temperature or lesion size has been reached. For the proposed MPC scheme in “Peak Temperature Control,” it is essential to estimate *T*_peak_ and α_rpe_ properly. The evaluation of the open-loop data showed that our approach enables the estimation of both in a reliable fashion. However, the open-loop data also showed that there is room for improvement by means of additional estimation of α_ch_. Future work could, for example, focus on experiment design techniques^38^^,^^39^ to design suitable (exciting enough) input signals (for open-loop experiments) and/or on using a laser with two different wavelengths to be able to reliably estimate both absorption parameters. In the following, we show and discuss the closed-loop results. We will also determine errors arising from the simplification of constant choroidal absorption in closed loop.

### Closed-Loop Results

**Figure 8.**
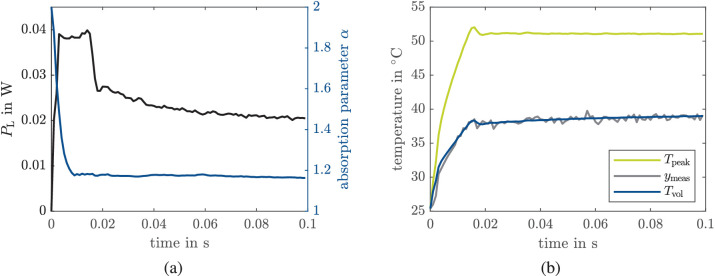
Closed-loop experiment. (a) Measured laser power *P*_L_ (black) and estimated absorption parameter α (blue) and (b) measured volume temperature *T*_meas_ (gray), estimated peak temperature *T*_peak_ (green), and estimated volume temperature *T*_vol_ (blue).

In this section, experimental results for closed loop integrating both the estimator and controller as described in “State and Parameter Estimation” and “Peak Temperature Control” (compare Fig. 3) are reported. Figure 8a shows a typical input signal with a laser power constraint of 40 mW and the estimation of the absorption parameter α. For the first two time steps, the laser power is set to fixed values of 20 mW and 25 mW to allow for software initialization that takes more than 1 ms. When approaching the aim temperature, the controller decreases the laser power. After a settling time of approx. 10 ms, the absorption parameter α stays nearly constant with slight changes due to measurement noise. In Figure 8b, the measured (volume) temperature and the estimated peak and volume temperature are shown. The aim temperature for the shown spot was 51°C. There is a small overshoot between 14 ms and 18 ms before *T*_aim_ is held until the end of the measurement.

An overview of the closed-loop control over all spots and different *T*_aim_ is shown in Table 2. We tested the closed-loop performance using two different laser power limits *P*_L, max_ = {30, 40} mW in the control design (compare Equation (3)). The average time *t*_{30, 40}_ until the (estimated) peak temperature reaches the aim temperature for the first time increases with increasing *T*_aim_. The rise times are different for the two laser power limits with longer rise times for *P*_L, max_ = 30 mW. The amount of spots where *T*_aim_ is not reached increases for higher *T*_aim_. In the case of *P*_L, max_ = 40 mW, the desired temperature is reached at all spots. Due to measurement noise and input noise (laser energy fluctuations), we count estimated peak temperatures that are at least 0.3°C above *T*_aim_ as an overshoot and define the overshoot as the difference between the estimated (maximum) peak temperature during the irradiation interval and the aim temperature (i.e., the desired peak temperature) *T*_os_ = *T*_peak_ − *T*_aim_. The duration of the overshoot *t*_os_ is the time until the peak temperature is (again) inside *T*_aim_ ± 0.3°C. We found that the duration of the overshoot is similar for all *T*_aim_. The intensity of the overshoot decreases with increasing *T*_aim_, whereas an overshoot occurs less frequently for higher *T*_aim_. We did not observe any constraint violation for the maximum allowed peak temperature (i.e., *T*_peak_ > *T*_max_ = *T*_aim_ + 2°C) or for the maximum laser power *P*_L, max_.

**Table 2.** Duration Until the Aim Temperature Is Reached, Number of Measurements Where the Aim Temperature Was Not Reached (Indicated by the Symbol <*T*_aim_), Maximum Overshoot, Duration of Overshoot, and Frequency for Different *T*_aim_

	Time Until *T*_aim_				Overshoot		
*T*_aim_	[*t*_30_, σ_30_] [ms]	<*T*_aim_ [%]	[*t*_40_, σ_40_] [ms]	<*T*_aim_ [%]	[*T*_os_, σ_os_] [°C]	[*t*_os_, σ_os_] [ms]	Freq. [%]
47	[13.4, 3.9]	0.0	[9.6, 0.5]	0.0	[1.2, 0.5]	[3.4, 0.5]	100
49	[33.5, 14.9]	0.0	[13.7, 1.9]	0.0	[0.9, 0.4]	[3.1, 0.9]	88.4
51	[43.6, 29.2]	3.9	[14.1, 2.9]	0.0	[1.1, 0.4]	[3.1, 0.8]	79.0
53	[60, 36.3]	31.0	[16.6, 3.8]	0.0	[1.0, 0.4]	[3.3, 0.5]	73.3
55	[66.3, 37.7]	50.0	[18.9, 5.5]	0.0	[0.9, 0.4]	[3.3, 0.5]	73.3
57	[71.0, 34.6]	57.1	[24.1, 6.2]	0.0	[0.8, 0.3]	[3.1, 0.8]	71.9
59	[76.2, 32.5]	64.0	[29.7, 9.0]	0.0	[0.7, 0.3]	[2.6, 1.4]	67.4
61	[85.5, 26.6]	76.2	[39.3, 13.7]	0.0	[0.7, 0.3]	[3.1, 0.9]	60.0

**Figure 9.**
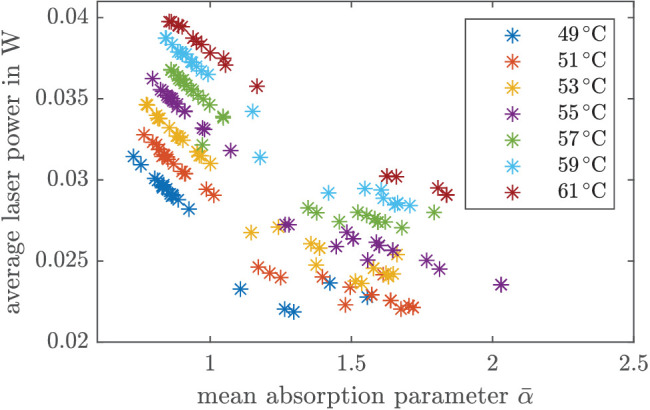
Estimated absorption parameter $\bar{\alpha }$ and the measured average laser power for a laser power limit of 40 mW and different aim temperatures from 49°C to 61°C and different eyes.

In Figure 9, the time average of the estimated absorption parameter $\bar{\alpha }$ and the average laser power ${\bar{P}}_{L}$ that was applied to the system within the irradiation time, that is, ${\bar{P}}_{L}=\frac{1}{100}\sum_{t=0\;s}^{0.099\;s}P_{L}\left(t\right)$, are shown for different aim temperatures from 49°C to 61°C. Each point represents a different spot. To avoid an influence of the settling time in the estimation (that is laser power dependent), we considered the last 50 ms for the calculation of $\bar{\alpha }$, that is, $\bar{\alpha }=\frac{1}{50}\sum_{t=0.05\;s}^{0.099\;s}\alpha \left(t\right)$. As can be seen from Figure 9, the smaller $\bar{\alpha }$, the greater is the average laser power that was applied to the system (for the same *T*_aim_). The higher *T*_aim_, the higher is the average power for similar absorption coefficients α.

**Figure 10.**
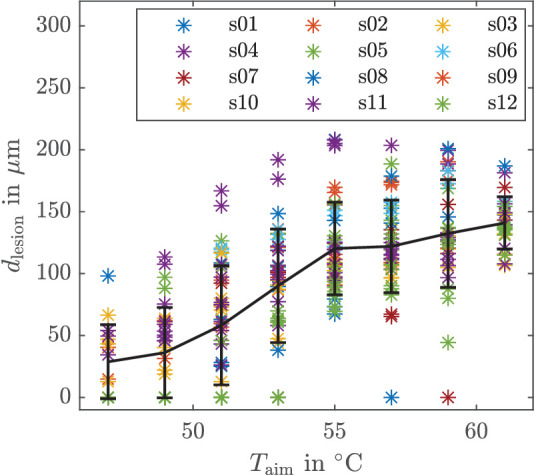
Desired aim temperature and resulting lesion diameter for all 12 eyes.

The diameter of the lesion *d*_lesion_ for the corresponding *T*_aim_ for all measurements where the aim temperature was reached is shown in Figure 10. The means over all lesions ${\bar{d}}_{\mathrm{lesion}}$ for a certain *T*_aim_ are depicted as a black line. The standard deviation for each *T*_aim_ is depicted as an error bar. The lesion diameter ${\bar{d}}_{\mathrm{lesion}}$ increases with increasing *T*_aim_. However, there are some outliers. A statistical evaluation of the lesion sizes is given in Table 3. With increasing *T*_aim_, the coefficient of variation $c=\sigma_{d}/{\bar{d}}_{\mathrm{lesion}}$ of the lesion diameter decreases.

**Table 3.** Mean Lesion Size and Deviation for Different *T*_aim_.

*T*_aim_	${\bar{d}}_{\mathrm{lesion}}$ [µm]	σ_d_ [µm]	Co. Var. *c*	No. Spots
47	29	30	1.03	16
49	36	37	1.03	30
51	58	48	0.83	44
53	90	46	0.51	44
55	120	37	0.31	44
57	122	37	0.30	41
59	132	44	0.33	33
61	141	21	0.15	23

### Discussion of Closed-Loop Experiments

In the following, we discuss the results obtained from our closed-loop experiments. We first comment on the controller performance in terms of the time to reach the aim temperature and overshoot (Table 2), as well as the applied average power (Fig. 9), before we analyze in more detail the main results from the calcein measurements in Table 3 and Figure 10.

From Table 2, it can be seen that the higher *T*_aim_, the longer it takes (in average) to reach *T*_aim_. This is to be expected due to active input (i.e., laser power) constraints that limit the speed of the temperature increase. Due to varying absorption, it is not possible to reach *T*_aim_ in all cases if the laser power is limited to 30  mW. From *T*_aim_ = 55°C on, for more than half of the measurements, the peak temperature is not reached. For *P*_L, max_ = 40 mW, the aim temperature is reached in all cases. For future experiments, the laser power limit can either be increased or the duration of the treatment can be extended. As a longer treatment is not in favor from an application point of view, *P*_L, max_ should be higher to guarantee that the aim temperature can be reached at each spot independent of the absorption. The average deviation σ_aim_ of the rise time *t*_{30, 40}_ over all spots (with the same *T*_aim_) increases also due to different absorption that determines *t*_{30, 40}_, that is, the time to reach *T*_aim_ (at bounded laser power). To lower the influence of the absorption on the time to reach *T*_aim_, a tracking control with a desired temperature rise profile *T*_aim_(*t*) could be used instead of the current setpoint control (i.e., *T*_aim_(*t*) = const∀*t* ⩾ 0). This might also have a positive effect on the deviation of the lesion size if the temperature rise is similar in all cases.

We observe an overshoot in more than 50% of the experiments (independent of *T*_aim_) with increasing frequency for smaller *T*_aim_. The duration of the overshoot is on average three time steps (i.e., 3 ms) independent of the aim temperature. The overshoot results from a time delay of two time steps that is present in the current setup and not from the control algorithm itself, that is, the solution of the optimal control problem Equation (3). The overshoot is in all cases below 2°C (no constraint violation), and the duration is short in comparison to the treatment time. Therefore, we do not expect a significant effect on the lesion size. Nevertheless, delay compensation MPC schemes^40^^,^^41^ could prevent an overshoot.

Figure 9 illustrates that the MPC behaves as expected in terms of optimal input calculation. The greater the absorption coefficient, the less average laser power is required to achieve the same aim temperature and thus the integral of the optimal laser power (i.e., the energy) obtained from solving Equation (3) is also smaller. Moreover, the average power increases with a higher aim temperature (and similar absorption coefficient). Both aspects underline that the MPC works well in combination with the EKF.

We now discuss the results obtained from the calcein measurements in Figure 10 and Table 3. First, observe that for a higher aim temperature, larger lesions are obtained, which is as desired and expected. Second, Figure 10 and Table 3 reveal the relation between a selected aim temperature and the resulting lesion size. This relation can be used to control the lesion size for mild coagulations via peak temperature control, which is a huge advantage over open-loop control. With known relation between peak temperature and lesion size, a safe and reliable treatment by means of closed-loop temperature control is made possible. For aim temperatures of around 60°C (compare the last two rows of Table 3), a similar mean lesion size can be seen as in the case of open-loop control with constant laser power of *P*_L_ = 30 mW (compare the second row of Table 1). However, when comparing the standard deviation (and hence the coefficient of variation) in these cases, one finds that the values are smaller for closed-loop compared to open-loop control. This means that as desired, the proposed closed-loop control scheme decreases the spread in the lesion size, that is, the lesion size depends less on the absorption than in open loop, which is of crucial importance for a reliable therapy, and underlines ones more a benefit of closed-loop control. We observe that there is still a significant spread in lesion size for a given aim temperature *T*_aim_. In the following, we discuss several possible explanations for this observation and ways to reduce the spread, if desired from a therapeutic perspective. Before that, we would like to point out that controlling the temperature in biological tissue is demanding due to its strong individual properties and that a certain spread in the lesion size will be inevitable.

The first possible reason for the spread in lesion size is the parameter drift (i.e., very slow convergence to a false parameter value) that was already discussed in “Discussion of Open-Loop Experiments.” To further evaluate this effect on the closed-loop behavior, we again performed additional simulations using the full-order model as the plant model (compare “Discussion of Open-Loop Experiments”).

**Figure 11.**
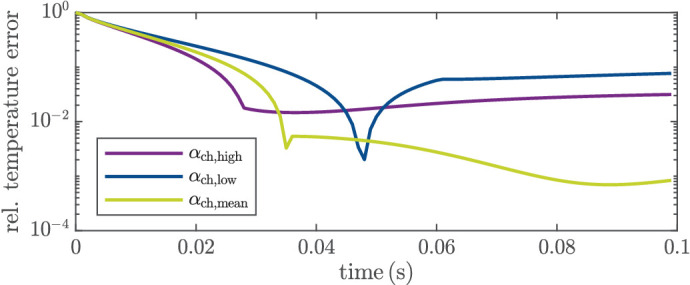
Relative error between the aim temperature and the peak temperature (of the plant model) in simulation, with mean choroidal absorption α_ch, mean_ = 0.1 (green), minimum absorption α_ch, low_ = 0.04 (blue), and maximum absorption α_ch, high_ = 0.15 (purple).

To this end, we changed the absorption in the plant model to α_ch, high_, α_ch, mean_, and α_ch, low_ (cf. “Discussion of Open-Loop Experiments” and Fig. 5). The (reduced) EKF model is the same in all three cases, with α_ch_ = α_ch, mean_. The EKF estimates the peak temperature to be the same as *T*_aim_ (after *T*_aim_ was reached for the first time). Due to the model mismatch caused by model reduction and falsely assumed choroidal absorption, the peak temperature of the (simulated) plant *T*_peak, plant_ is different. Figure 11 shows the relative error between *T*_aim_ and *T*_peak, plant_ over time, that is, e rel (t)=|T aim -T peak , plant (t)|T aim  for *T*_aim_ = 55°C. The error is the largest at the beginning, since the sample is not yet heated. For a correctly assumed choroidal absorption, the error is only caused by a model reduction error and a state estimation error. Hence, it is the smallest with *e*_rel_ = 0.0008 after *t*_e_ = 100 ms, that is, *e*_mean_(*t*_e_) = *T*_aim_ − *T*_peak, plant_(*t*_e_) = −0.02°C. For a higher choroidal absorption, α_rpe_ is overestimated and *T*_aim_ is not reached at all. Because of the parameter drift, the estimation error of the peak temperature increases also over time, as shown in Figure 11. This leads to an increasing offset (after the initial temperature rise) between the estimated and the real (simulated) peak temperature with *e*_rel_ = 0.03 after 100 ms, that is, *e*_high_(*t*_e_) = 0.9°C. In the case of a smaller choroidal absorption, the peak temperature is underestimated, which leads to a peak temperature above *T*_aim_. After 100 ms, the absolute error is slightly above the maximum allowed temperature *T*_max_ in Equation (3), that is, *T*_peak, plant_ > *T*_max_ with *e*_low_(*t*_e_) = −2.3°C. Keep in mind that these (rather large) errors are the worst-case scenarios in terms of changing choroidal absorption, meaning in 95% of the case study in Schaller et al.,^20^ the error is smaller (for the same aim temperature). Nevertheless, this could be one reason why the deviation in the lesion diameter in Table 3 for the same *T*_aim_ is higher than we expected by closed-loop control.

A second reason of error could be that the laser profile changes if the focus of the beam is not exactly at the RPE. If so, the laser beam cannot be considered a so-called *tophat* beam with a constant and uniform intensity but rather as a Gaussian beam. Further intensity modulations due to coherent mode matching in the multimode fiber are not considered, which can lead to strong intensity peaks at the irradiated spot. The influence of the beam profile might be stronger for temperatures close to the coagulation threshold, where the profile is decisive for (visible) damage.

A third reason for the spread in lesion size could be inaccuracies in the temperature measurement due to coagulation. Each laser pulse induces an expansion of the tissue that yields a pressure wave that is measured.^11^ During coagulation, the expansion properties of the tissue (especially the Grüneisen parameter) change. In addition, light scattering takes place in coagulated tissue, which has an influence on the light distribution and thus absorption profile at the irradiated site. Both changing tissue parameters and the onset of light scattering override the temperature calibration and lead to a falsified volume temperature.^42^ Although the influence on the volume temperature is small for mild coagulations,^15^ it can still have an effect on the peak temperature estimation. Therefore, our method is especially suitable for subdamaging hyperthermia treatments and mild coagulations to a certain extent. In the case of strong coagulation, no reliable determination of the temperature can be guaranteed, and therefore no reliable feedback for the estimator/controller can be expected. However, the associated sudden change in the pressure signal at the onset of coagulation could also be used to stop the treatment at an early stage to prevent a larger coagulation or even tissue bleeding and ruptures.

Finally, another reason could also be that melanin clusters occur at one or another spot. These clusters can lead to a strong deviation in the absorption within the irradiation area of one spot or in different layers at one spot. This can favor a nonuniform heating, which cannot be taken into account by our model Equation (2).

We now briefly discuss possible adaptations in the setting and estimation algorithm to improve the accuracy of the developed closed-loop controller in future experiments (i.e., to reduce the still existing spread in lesion size for a given aim temperature). First, optical coherence tomography (OCT) can be used to monitor the heat diffusion online as in Müller et al.^43^ and help to verify/support our conjectures made in this section. Also, as already discussed in “Discussion of Open-Loop Experiments”, a second wavelength could be used to gain more information about a depth-dependent absorption and to facilitate the estimation of the choroidal absorption. Closed-loop estimation for more than one parameter can be implemented by means of sensitivity updates.^44^

## Limitations

We have shown that model predictive control has the potential to improve retinal laser treatments. Our experiments were conducted on ex vivo explants of porcine eyes, which are often used to study laser impacts on the choroid/retinal junction as their morphology is similar to that of humans.^45^ Therefore, we expect similar results with human eyes. However, by irradiating RPE/choroid/sclera explants instead of living eyes, some limitations need to be considered. First of all, heat convection due to blood flow is not included in the modeling. This can be done by an additional heat sink; see Sandeau et al.,^46^ where a perfusion term is added to the heat diffusion equation. However, it was found that heat loss by blood perfusion can be neglected for such a short irradiation time of 100 ms.^47^ If the tissue is irradiated for several seconds, perfusion cannot be neglected, as shown for living and dead rabbits in Hermann et al.^48^ Hence, the model needs to be adapted when it comes to such long irradiation times. Independent of in vivo or ex vivo irradiation, the absence of cornea, lens, and vitreous body simplifies the parameter identification. In particular, scattering in the lens affects the amount of light that reaches the eye fundus. As scattering varies individually and depends, among others things, on the age of the patient,^49^ it may be necessary to identify an additional transmission factor that scales the laser power reaching the eye fundus. Since this parameter can be assumed to remain constant throughout the eye, it can be identified initially and then kept constant during treatment at different spots. Concerning the explant model in this work, we have assumed that the density, thermal conductivity, and heat capacity are equal to those of water as it is the main component of the tissue. However, the model could be refined by including layer-dependent parameters in following versions.

There are also some inevitable methodological limitations. First, there might be local laser intensity modulations at the irradiated spot. This is caused by different propagation modes of the coherent light in multimode optical fibers, which leads to a phase shift and, thus, to an inhomogeneous energy distribution. Second, within our measurement technique, we assume that the thermal expansion coefficient is independent of the tissue. The underlying reasoning is the following: as mainly the RPE and the choroid influence the thermoelastic response, this error should be small. Moreover, the focus of the laser beam at the eye fundus depends on the proper focusing by the ophthalmologist and is most likely not always the ideal top hat. This can lead to a broader spot size and to a (super) Gaussian beam profile instead of a tophat beam as considered in the model.

A key issue with regard to the translation into clinical practice are involuntary eye movements such as drift, saccades, and tremor. If the eye moves during laser light application, a line will be irradiated. This movement cannot be captured by our reduced order models, and thus it leads to errors in estimation and control. However, there are ways to prevent over- or undertreatment despite eye movements. If the eye moves, there will be a recognizable change in the temperature course, as shown by Brinkmann et al.^50^ during temperature-controlled patient treatment by means of a PID controller. This change can be detected by sudden shifts in the parameter estimation. Thus, the EKF can be utilized as a fault detector. The treatment could then simply be stopped or paused until the remaining heat has been dissipated and the spot will then be reirradiated.

Last but not least, the temperature measurement technique is only suitable for subdamaging treatments or mild coagulations, as pointed out before. However, our control approach could also be used for strong coagulations in an open-loop fashion, that is, we use closed-loop control for estimation and control until a certain temperature is reached and then use the solution of the optimal control problem (with a larger horizon length) until the end of the treatment. Applying MPC in open loop for strong coagulations is part of future work.

## Conclusions

The aim of this first (extensive) experimental study for model predictive temperature-controlled retinal laser treatments was to investigate if and how well the proposed control strategy works by evaluating open-loop data, closed-loop data, and calcein measurements with respect to damage ranges depending on the temperature. We have shown that we are able to estimate and control the peak temperature. The evaluation of the fluorescence images showed that the lesion size increases with increasing peak temperature. The spread in the lesion size was smaller for closed-loop than for open-loop control. This demonstrates that the proposed EKF-MPC scheme can lead to a safer treatment in terms of under- or overtreatment for sub-damaging treatments or mild coagulations (uniform lesion size). However, the deviation in the lesion size in closed loop was unexpectedly high and leaves room for improvement. Especially the estimation of the choroidal absorption in addition to the RPE absorption seems to be promising to improve the estimation of the peak temperature and hence to reduce the deviation in lesion size. To this end, a second wavelength can be used to ease the estimation of the choroid absorption. This is part of future work.

Furthermore, higher laser powers can increase the number of successful treatments where the desired aim temperature is reached but must be weighted with regard to safety. A tracking control (i.e., a time-varying aim temperature profile) in combination with higher laser power can reduce the strong deviation in the rise times (due to absorption) that enables a better comparability of the results. To examine further influences on the lesion size like, for instance, the laser beam profile or melanin clusters, experiments in combination with OCT that can visualize the heat diffusion online are part of future work.
